# Editorial: Hybrid Intelligent Algorithms Based Learning, Optimization, and Application to Autonomic Control Systems

**DOI:** 10.3389/fnins.2019.01090

**Published:** 2019-10-11

**Authors:** Yanzheng Zhu, Hak-Keung Lam, Ting Yang, Zhixiong Zhong, Sabri Arik

**Affiliations:** ^1^College of Mechanical Engineering and Automation, Huaqiao University, Xiamen, China; ^2^Department of Informatics, King's College London, London, United Kingdom; ^3^School of Automation, Northwestern Polytechnical University, Xi'an, China; ^4^College of Computer and Control Engineering, Minjiang University, Fuzhou, China; ^5^Department of Computer Engineering, Faculty of Engineering, Istanbul University-Cerrahpasa, Istanbul, Turkey

**Keywords:** artificial intelligence, autonomous systems, intelligent control, machine learning, optimization

With the rapid rise of artificial intelligence, a large amount of intelligent techniques, including neural networks (Zhang et al., 2015), fuzzy logic (Zhong et al., 2017), and genetic algorithms (Rovithakis et al., 2004) have been broadly applied to various fields, such as chemical process, robotics, mechanical engineering, etc. In biological systems, the neural networks usually contain a finite set of modes that switch in accordance with internal evolution and external stimulation, and such switching can often be represented as stochastic (Zhang et al., 2016) or even non-deterministic form (Yang and Zheng, 2018). Recent novel developments in the system and control community on control and filtering of intelligent systems with some hybrid switching characteristics. However, the practical applications in the areas of tele-medicine, disease treatment, and healthcare are lacking due largely to the limitations of existing hybrid intelligent systems. It is also difficult and challenging to implant these hybrid intelligent algorithms into the process of existing manufacturing facilities and equipment research and development.

Recently, autonomous control (Antsaklis et al., 1991; Pachter and Chandler, 1998)—based on the mode of operation of the autonomic nervous system-has emerged with the advent of artificial intelligence, and an ever-increasing demand has been placed by users in different fields (Isakhani et al., 2018; Shen et al., 2018). It is expected that the advanced intelligent algorithms can be fitted into the learning, optimization and control design to improve autonomous ability. Also, exploration of the novel communication mechanisms between autonomous systems and other regulatory systems is very welcome, building on the existing approaches on networked control systems with communication constraints (Heemels et al., 2010). The current Research Topic provides a useful overview of hybrid intelligent algorithm-based learning, optimization and the applications of these new autonomous control strategies.

Wang et al. investigate the stability and stabilization control problem for non-linear T-S fuzzy sampled-data systems under time-varying sampling intervals, with and without quantized states. A new Lyapunov-Krasoskii functional (LKF) named discontinuous LKF is constructed, such that the LKF is not necessary to be positive all the time, but only positive at the sampling time. By using the proposed discontinuous LKF approach and free-matrix-based integral inequality boundary processing technique, stability conditions that are less conservative are obtained for T-S fuzzy systems with and without sampled-data quantized states, and the required sampled-data controllers are designed simultaneously. The simulation results show that the maximum sampling interval of T-S fuzzy sampling-data systems with and without quantized states are both larger than the existing results.

Zhang et al. establish the periodic event-triggered control (PETC) scheme of robust *H*_∞_ filtering for a class of uncertain discrete-time Takagi-Sugeno (T-S) fuzzy systems, where the sample time is assumed to be a constant. Two frameworks are introduced based on perturbed linear and piecewise linear systems to analyze the filtering problems of the PETC strategy. Sufficient conditions for the existence of a robust *H*_∞_ filter are derived in the form of matrix inequalities. The effectiveness of the proposed approach is testified by using a simulation example.

Guan et al. propose a robust adaptive recurrent cerebellar model articulation controller (RCMAC) for non-linear MIMO systems, where the GPSO-based RCMAC with the adaptive law is used for updating parameters, and the learning rates can be optimized to best value based on the GPSO algorithm. It has been shown that the proposed robust controller can be designed to compensate for the difference between adaptive RCMAC and ideal controller.

Zhao et al. present the wavelet fuzzy brain emotional learning controller (WFBELC) model for the uncertainty of the MIMO non-linear systems based on the wavelet theory, type-1 fuzzy inference and the BEL algorithm. The WFBELC is used as the main tracking controller for a MIMO uncertain non-linear system and the robust compensation controller is used as a compensator. It has been shown that this proposed WFBELC model can effectively obtain satisfactory control capability with better transient responses and smaller error values, compared to the fuzzy cerebellar model articulation controller (FCMAC) design scheme and the brain emotional learning controller (BELC) design scheme.

Bi et al. introduce a novel model called the Genetic-Evolutionary Random Support Vector Machine Cluster (GE-RSVMC) to classify individuals with Asperger syndrome and neurotypical individuals, and search for lesions within the brain. The model innovatively integrates the methods of cluster and genetic evolution to improve the performance of the model. It has been shown that the classification accuracy of the model reaches 97.5% and identifies brain regions showing significant differences. The proposed method also provides a new perspective for the diagnosis and treatment of Asperger syndrome.

## Author Contributions

All authors listed have made a substantial, direct and intellectual contribution to the work, and approved it for publication.

### Conflict of Interest

The authors declare that the research was conducted in the absence of any commercial or financial relationships that could be construed as a potential conflict of interest.
